# ACM-Assessor: An Artificial Intelligence System for Assessing Angle Closure Mechanisms in Ultrasound Biomicroscopy

**DOI:** 10.3390/bioengineering12040415

**Published:** 2025-04-14

**Authors:** Yuyu Cong, Weiyan Jiang, Zehua Dong, Jian Zhu, Yuanhao Yang, Yujin Wang, Qian Deng, Yulin Yan, Jiewen Mao, Xiaoshuo Shi, Jiali Pan, Zixian Yang, Yingli Wang, Juntao Fang, Biqing Zheng, Yanning Yang

**Affiliations:** 1 The Department of Ophthalmology, Renmin Hospital of Wuhan University, Wuhan 430061, China; cyyoo99@163.com (Y.C.); jiangweiyanoph@163.com (W.J.); zhujian0827@sina.com (J.Z.); wangyj@whu.edu.cn (Y.W.); whudq501@whu.edu.cn (Q.D.); ophyanyulin@gmail.com (Y.Y.); ophmjw@163.com (J.M.); 2018305231079@whu.edu.cn (X.S.); 2018305232047@whu.edu.cn (J.P.); 18702728360@163.com (Z.Y.); whuwangyingli@163.com (Y.W.); fjt1148809308@163.com (J.F.); 2 The Department of Gastroenterology, Renmin Hospital of Wuhan University, Wuhan 430061, China; d_dongzehua@163.com; 3 The Department of Comprehensive Plastic Surgery, Plastic Surgery Hospital, Chinese Academy of Medical Sciences and Peking Union Medical College, Beijing 100144, China; yangyuanhao0202@163.com; 4 The School of Resources and Environmental Sciences, Wuhan University, Wuhan 430061, China; 21cnzbq@21cn.com

**Keywords:** artificial intelligence, ultrasound biomicroscopy, primary angle-closure glaucoma, angle closure mechanisms

## Abstract

Primary angle-closure glaucoma (PACG), characterized by angle closure (AC) with insidious and irreversible progression, requires precise assessment of AC mechanisms for accurate diagnosis and treatment. This study developed an artificial intelligence system, ACM-Assessor, to evaluate AC mechanisms in ultrasound biomicroscopy (UBM) images. A dataset of 8482 UBM images from 1160 patients was retrospectively collected. ACM-Assessor comprises models for pixel-to-physical spacing conversion, anterior chamber angle boundary segmentation, and scleral spur localization, along with three binary classification models to assess pupillary block (PB), thick peripheral iris (TPI), and anteriorly located ciliary body (ALCB). The integrated assessment model classifies AC mechanisms into pure PB, pure non-PB, multiple mechanisms (MM), and others. ACM-Assessor’s evaluation encompassed external testing (2266 images), human–machine competition and assisting beginners’ assessment (an independent test set of 436 images). ACM-Assessor achieved accuracies of 0.924 (PB), 0.925 (TPI), 0.947 (ALCB), and 0.839 (integrated assessment). In man–machine comparisons, the system’s accuracy was comparable to experts (*p* > 0.05). With model assistance, beginners’ accuracy improved by 0.117 for binary classification and 0.219 for integrated assessment. ACM-Assessor demonstrates expert-level accuracy and enhances beginners’ learning in UBM analysis.

## 1. Introduction

Glaucoma is the leading eye disease causing blindness globally, and primary angle-closure glaucoma (PACG) is the leading type of glaucoma in Asia, especially in China [1,2,3]. It is estimated that there will be approximately 110 million glaucoma patients worldwide by 2040, posing a serious threat to human visual health [4,5].

Progression to PACG generally follows two precursor stages: primary angle-closure suspect (PACS) and primary angle closure (PAC), collectively termed primary angle-closure disease (PACD) [6]. The irreversible nature of PACG underscores the need for early detection and treatment. Angle closure (AC) mechanisms include pupillary block (PB) and non-PB, such as thick peripheral iris (TPI) and an anteriorly located ciliary body (ALCB). In most cases, AC results from the combined effects of both PB and non-PB, known as ‘multiple mechanism’ (MM) [7,8]. Studies show therapeutic efficacy varies across PACD patients based on AC mechanisms [9,10,11]. Accurate identification of AC mechanisms enables personalized treatment plans, advancing individualized precision therapy [12,13]. However, clear quantitative criteria are lacking, leaving assessments reliant on clinical experience.

Gonioscopy is the gold standard for anterior chamber angle (ACA) assessment, but limitations—such as its inability to visualize post-iris structures, technical difficulty, and invasiveness—hinder its broader application [14,15]. In contrast, ultrasound biomicroscopy (UBM) uses sound waves to penetrate the pigment epithelium, enabling visualization of post-iris structures like the suspensory ligament, posterior chamber, and ciliary body, thus enhancing AC mechanism insights. However, UBM image interpretation remains subjective, technically challenging, and time-intensive [14]. This underscores the urgent need for advanced tools to improve UBM image analysis.

Artificial intelligence (AI) has been increasingly applied in ophthalmic disease diagnosis [16,17], with recent models predicting AC mechanisms using anterior segment optical coherence tomography (AS-OCT) images [18,19,20,21,22]. However, current AS-OCT-based approaches face critical limitations, including insufficient external validation, an inability to assess the ciliary body, and a limited capacity to detect MM in complex cases, that impede their broad clinical adoption. Notably, despite UBM’s unique advantages in assessing AC mechanisms, the development of AI systems for AC mechanism assessment using UBM remains significantly underexplored. Building on our previous research, where we developed models for automated ACA structure segmentation in UBM images [23], this study aims to advance the automatic recognition of AC mechanisms in UBM images, exploring its clinical applications and potential benefits.

## 2. Materials and Methods

### 2.1. Datasets and Labeling

This study retrospectively collected images of PACD patients who underwent UBM (SW-2100; Tianjin Suowei Co., Ltd., Tianjin, China) at the Renmin Hospital of Wuhan University (RHWU; Wuhan, China) and Huangshi Aier Eye Hospital (HAEH; Huangshi, China) between August 2022 and May 2024. All images were acquired by a senior ophthalmic technician with over 20 years of experience, following strict specifications. The collected images have dimensions of 1024 × 655 pixels. In this study, all images were obtained in half-frame mode (ultrasound probe frequency, 50 MHz; scanning range, 9.75 mm × 6.00 mm) or panorama mode (ultrasound probe frequency, 35 MHz; scanning range, 15.50 mm × 9.50 mm). In clinical practice, during the acquisition of UBM images, four UBM images are typically captured for each eye, corresponding to the positions at 3, 6, 9, and 12 o’clock. For each patient, one image per quadrant is retained for analysis. The study was approved by the Ethics Committee of RHWU (Approval No. WDRY-2022-K109), and the study was undertaken in accordance with the Declaration of Helsinki. As this study was retrospective and utilized desensitized UBM images, no informed consent was required.

**Figure 1:**
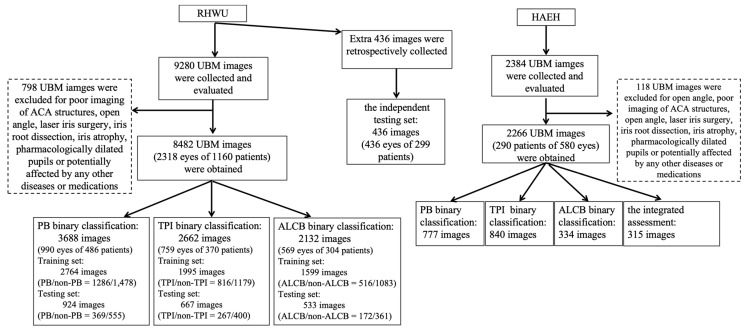
Flowchart of the model development and validation. RHWU, Renmin Hospital of Wuhan University; HAEH, Huangshi Aier Eye Hospital; UBM, ultrasound biomicroscopy; ACA, anterior chamber angle; PB, pupillary block; TPI, thick peripheral iris; ALCB, anterior located ciliary body.

Professional ophthalmologists screened the collected images, and each image contained only one side of the ACA. Images were excluded based on: (1) poor quality (e.g., device malfunction, operational errors, motion artifacts, or insufficient contrast); (2) incomplete ACA structures, such as acquisition errors in ciliary process positioning; (3) secondary structural alterations, such as laser iris surgery, pharmacologically dilated pupils, iridodialysis and uveitis; and (4) open-angle configurations lacking iridotrabecular contact [24]. A total of 8482 UBM images from 2318 eyes of 1160 patients were obtained, and the number of UBM images used for PB, TPI, and ALCB binary classification models was 3688 from 990 eyes of 486 patients, 2662 from 759 eyes of 370 patients, and 2132 from 569 eyes of 304 patients, respectively. The training and testing sets were randomly set up according to the ratio of 3:1. Images from the same patient do not appear in both the training and testing sets. An additional 436 images from 436 eyes of 299 patients were utilized as the independent testing set for the integrated assessment model, including human–machine comparison and assistance for beginners. Additionally, 2266 UBM images from HAEH were selected as the external testing set. The breakdown of data for these different datasets is illustrated in Figure 1.

Each image underwent initial labeling by two senior ophthalmologists with over 10 years of clinical experience. In cases of disagreement, a third ophthalmologist with more extensive experience adjudicated the final labeling. The labeling process involved identifying and assessing the presence or absence of PB, TPI, and ALCB in AC mechanisms, as well as the integrated assessment (Figures S1–S4). The ophthalmologist performed the labeling based on established definitions [7,8,12,25,26] and their extensive clinical experience. Specifically, in this study, the presence of PB indicated that PB was one mechanism of AC, regardless of whether other AC mechanisms were also present in the image; the same criterion applied to TPI and ALCB. Building upon the binary classifications of PB, TPI, and ALCB, the integrated assessment further categorized AC mechanisms into:

Pure PB: only PB, non-TPI and non-ALCB;

Pure non-PB: only TPI or ALCB, non-PB;

MM: PB and (TPI or/and ALCB);

Others: non-PB, non-ALCB and non-TPI.

### 2.2. Development of the Model

**Figure 2:**
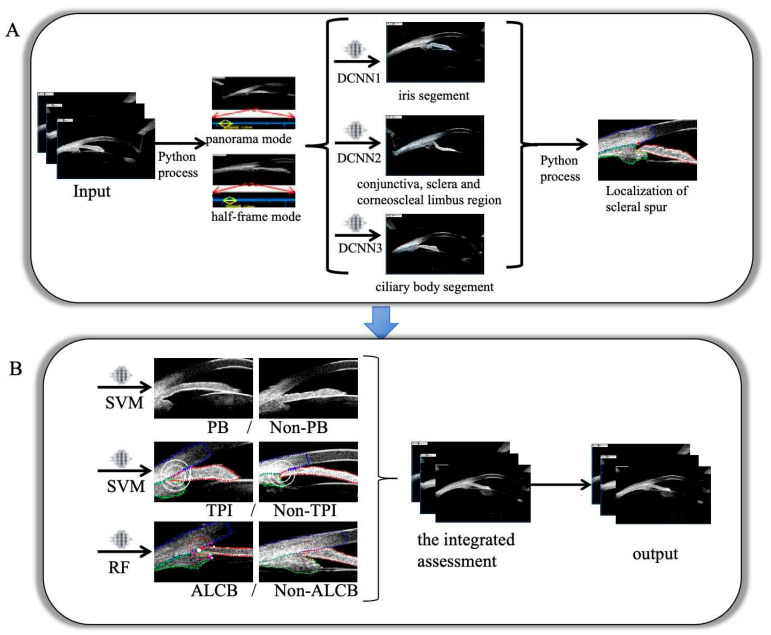
The flowchart of the model. Images were imported into the proposed architectures. (**A**) Image preprocessing using the pre-study model: conversion of image pixel values to numerical values using Python program, identification of ACA structures by DCNN1~3, and localization of scleral spur using Python program. (**B**) Models were constructed using the SVM, SVM and RF algorithms, respectively, to identify the presence or absence of PB, TPI, and ALCB. The integrated assessment is then performed. Eventually, the output results. DCNN, deep convolution neural network; PB, pupillary block; TPI, thick peripheral iris; ALCB, anterior located ciliary body; SVM, support vector machine; RF, random forest.

The preprocessing of UBM images was performed using the pre-published models by our team [23], as illustrated in Figure 2A. First, the pixel values of the half-frame mode and panorama mode images were converted to numerical values. Then, the ACA structure area was automatically segmented into the iris area, sclera area, and ciliary body area using the UNet++ network. A Python image processing program was employed to localize the scleral spur automatically.

After preprocessing UBM images, classification criteria were encoded in Python. (i) For PB detection, the algorithm used iris root insertion and curvature assessments, as in previous models [23]. (ii) For TPI detection, circles with radii of 500 and 750 µm centered on the scleral spur intersected the iris, and distances between these intersections were measured. Another 500 µm radius circle around the iris root was similarly drawn and measured. (iii) For ALCB detection, the length of contact between the anterior ciliary body and the posterior iris was measured, and its ratio to the total length of the anterior ciliary body surface was calculated. Processed UBM images and results served as inputs for algorithms like extreme gradient boosting (XGB), random forest (RF), gradient boosting decision tree (GBDT), support vector machine (SVM), and logistic regression (LR), which were obtained from repositories, such as XGBoost and scikit-learn. The best-performing models were evaluated using the testing sets (Figure 2B).

The integrated assessment (Figure 2B): Using the three developed models, the input UBM images were categorized into AC mechanisms: pure PB, pure non-PB, MM, or others [7,8]. These classifications were validated using the independent testing set. Figure 2 illustrates a detailed workflow of the model.

The algorithms were implemented in Python 3.6.5. The deep learning models were trained on the Keras 2.2.5 (https://github.com/keras-team/keras, accessed on 4 March 2023) framework with TensorFlow 1.12.2 (https://github.com/tensorflow/tensorflow, accessed on 4 March 2023) serving as the computational backend. Training was performed on a server equipped with a NVIDIA GeForce GTX 1080 (8GB GPU memory).

### 2.3. Evaluation and Experiments

The internal testing sets (RHWU) and the external testing sets (HAEH) were utilized to evaluate the models’ performance in predicting PB, TPI, ALCB, and integrated assessment.

We used 436 UBM images from an independent testing set to compare model performance with that of three experts, each with over 20 years of UBM experience and uninvolved in annotation. The experts evaluated the PB, TPI, ALCB, and integrated assessment, with a staff member recording their results and time taken. The models analyzed the same elements, and their accuracy and evaluation time were then compared to those of the experts.

To assess the models’ effectiveness as a diagnostic and training tool, eight beginners (less than 3 years’ experience with UBM) independently diagnosed 436 images without model assistance. After an 8-week washout, they reassessed the same images with model-aided diagnostic labels as reference. A staff member recorded the results of both assessments, allowing for accuracy comparisons.

Accuracy, sensitivity, specificity, positive predictive value (PPV), negative predictive value (NPV), F1 score, Matthews correlation coefficient (MCC), receiver operating characteristic (ROC) curves, and area under the curve (AUC) were utilized to assess the performance of the models. A chi-square (χ^2^) test was performed to evaluate the difference in accuracy between the model and the ophthalmologists. The inter-rater agreement among ophthalmologists was assessed using Cohen’s kappa coefficient. SPSS 27.0 software (IBM, Armonk, NY, USA) was used for statistical analysis.

## 3. Results

### 3.1. Results in Both Internal and External Test Datasets

**Figure 3:**
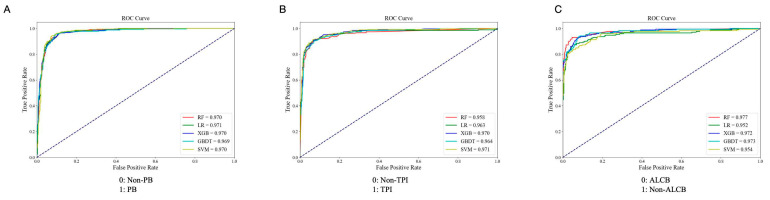
ROC curve diagram of classification models with the different algorithms. (**A**) ROC curve of PB/Non-PB; (**B**) ROC curve of TPI/Non-TPI; (**C**) ROC curve of ALCB/Non-ALCB. PB, pupillary block; TPI, thick peripheral iris; ALCB, anterior located ciliary body; RF, random forest; GBDT, gradient boosting decision tree; XGB, extreme gradient boosting; SVM, support vector machine; LR, logistic regression.

**Figure 4:**
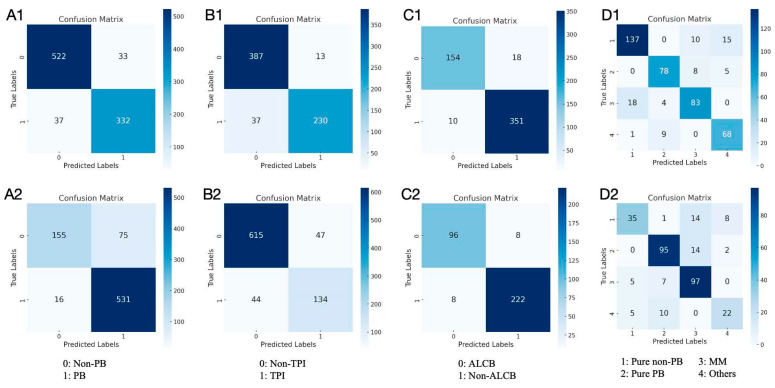
Confusion matrix of classification models with the best performing algorithm. (**A1**–**D1**) Confusion matrix of internal testing sets. (**A2**–**D2**) Confusion matrix of external testing sets. PB, pupillary block; TPI, thick peripheral iris; ALCB, anterior located ciliary body; MM, multiple mechanisms.

This study evaluated various classification models using the manual classifications of senior ophthalmologists as the gold standard. Inter-expert agreement assessments during the annotation process are shown in Table S3. SVM, SVM, and RF algorithms achieved the highest MCC, F1 score, accuracy, and sensitivity in the PB, TPI, and ALCB binary classification models, respectively. Therefore, these algorithms were chosen to construct the final models. Table 1 compares models using different algorithms. In the internal testing set, the PB, TPI, and ALCB models achieved high accuracy: 0.924 (95% CI = 0.905–0.940), 0.925 (95% CI = 0.902–0.943), and 0.947 (95% CI = 0.924–0.964), with AUC values over 0.95 (Figure 3). External test accuracy rates were 0.883 (95% CI = 0.858–0.904), 0.892 (95% CI = 0.868–0.912), and 0.952 (95% CI = 0.922–0.972), respectively. Additionally, the integrated assessment model reached 0.839 (95% CI = 0.801–0.872) accuracy in the internal test and 0.739 (95% CI = 0.688–0.784) in the external test. Confusion matrices for each model in both datasets are shown in Figure 4.

### 3.2. Performance of the Models and Experts

The models and three experts evaluated 436 UBM images from the independent testing set, with the results shown in Table 2. The mean accuracy for identifying PB was 0.867 (95% CI = 0.831–0.897) for the model and 0.860 (95% CI = 0.823–0.891) for experts. For TPI, the model achieved 0.828 (95% CI = 0.789–0.862) accuracy, compared to 0.839 (95% CI = 0.801–0.872) by experts. For ALCB, the model’s accuracy was 0.897 (95% CI = 0.863–0.923), while the experts’ accuracy was 0.878 (95% CI = 0.843–0.907). In the integrated assessment, model accuracy was 0.837 (95% CI = 0.798–0.870), compared to 0.791 (95% CI = 0.750–0.828) for experts, with no significant accuracy differences (*p* > 0.05). The models completed the evaluation in 784.80 s, approximately four times faster than the experts, who took 3020.39 s (Table 2).

### 3.3. Comparison of the Performance of Beginners with and Without the Model Assistance

**Figure 5:**
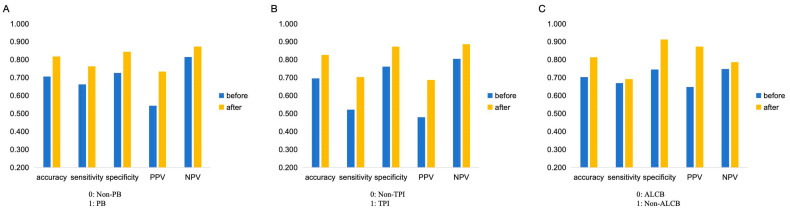
The average performance changes in binary classification by the eight beginners before and after model assistance. (**A**) The average performance of eight beginners in PB/Non-PB; (**B**) The average performance of eight beginners in TPI/Non-TPI; (**C**) The average performance of eight beginners in ALCB/Non-ALCB. PB, pupillary block; TPI, thick peripheral iris; ALCB, anterior located ciliary body; RF, random forest; GBDT, gradient boosting decision tree; XGB, extreme gradient boosting; SVM, support vector machines; LR, logistic regression.

Before model assistance, the mean accuracies of the eight beginners in identifying PB, TPI, ALCB, and integrated assessment were 0.706, 0.704, 0.696, and 0.432, respectively. After model assistance, these accuracies improved to 0.817, 0.814, 0.827, and 0.651, respectively, showing significant improvement (*p* < 0.05). Figure 5 and Table S1 illustrate the average performance changes in binary classification, and Table S2 shows accuracy changes in integrated assessment before and after model assistance.

## 4. Discussion

Approximately half of PACD cases in China are due to MM, one-third to pure PB, and less than 10% to pure non-PB [8]. Studies have shown that the majority mechanism of acute AC and its contralateral eye is PB, while non-acute AC is predominantly caused by non-PB or MM [27,28]. If left untreated, acute AC can lead to significant vision loss. Therefore, screening for AC mechanisms in patients with PACS is crucial for the early prevention of acute AC. Unlike primary open-angle glaucoma (POAG), PACG can often be partially prevented. Effective PACG management requires not only reducing intraocular pressure (IOP) but also addressing AC dynamics and drainage mechanisms unique to PACG [29,30]. For example, laser peripheral iridotomy (LPI) addresses PB but is unsuitable for non-PB, where non-PB may require pupillary reduction or argon LPI (ALPI) to widen the ACA [24,29,30]. Thus, precise identification of AC mechanisms is essential for effective PACD treatment.

In this study, the ACM-Assessor was developed to evaluate AC mechanisms in UBM images using deep learning, machine learning, and Python. The design includes models for pixel-to-physical spacing conversion, ACA boundary segmentation, scleral spur localization [23], and three binary classification models. This comprehensive approach accurately captures critical UBM image features, establishing a strong foundation for AC mechanism evaluation. The binary classification models for PB, TPI, and ALCB achieved impressive accuracy exceeding 0.920. These results affirm the suitability of the proposed classification models, which utilize feature extraction and custom classifiers. Python processing integrates feature extraction expertise—for example, PB is identified by factors like posterior chamber pressure and iris curvature [23], TPI by peripheral iris thickness [7,8,25], and ALCB by iris–ciliary body contact extent [7,8]. This approach enhances both model performance and scientific reliability.

AI has recently contributed significantly to ophthalmology, yet applications in UBM image recognition remain relatively underexplored, primarily addressing tasks such as scleral spur localization [31], ACA classification [32,33], and the ACA measurement [32,33,34]. Assessment systems targeting AC mechanisms have also been developed, mainly utilizing AS-OCT images [18,19]. However, these approaches often require manual scleral spur identification, introducing subjectivity and variability into semiautomated methods. Furthermore, most prior studies focus on a single predominant mechanism per image, even though AC often results from MM [7,8].

Compared to previous studies, ACM-Assessor offers key advantages. It is the first system for automating AC mechanisms assessment using UBM rather than AS-OCT, requiring only a UBM image input without manual localization of structures like the scleral spur, thus minimizing subjective errors. Unlike prior studies [18,19,20,21,22] that identified one single AC mechanism per image, ACM-Assessor identifies MM within one single image, addressing the frequent coexistence of MM in AC patients [7,8]. This classification approach aligns with clinical challenges, supporting diagnosis and treatment decisions. Additionally, it uniquely identifies ALCB-related mechanisms, enabled by UBM’s superior visualization of the ciliary body [15]. Furthermore, integrating the models of this study with our previous research [23] enables the initial screening of patients with AC in primary healthcare settings. Subsequently, precise identification of the underlying mechanisms of AC in these patients can be achieved, thereby significantly enhancing the practical utility of the proposed approach.

Compared with experts, ACM-Assessor demonstrated equivalent identification accuracy (*p* > 0.05) and outperformed average expert accuracy in identifying PB, ALCB, and integrated assessment. Moreover, the system operates four times faster than experts. The inability of experts to achieve 100% accuracy may arise from visual fatigue during extended readings and the subjective nature inherent in UBM image interpretation among different experts. The fully automated recognition capability of the proposed models could significantly eliminate assessment errors due to subjectivity and reduce clinicians’ workload.

Beginners initially achieved an accuracy of 0.702 in binary classification and 0.432 in the integrated assessment, underscoring the challenge of applying theoretical knowledge to practical AC mechanisms identification. With model assistance, their binary classification accuracy improved to 0.819 (a 0.117 increase), and their integrated assessment accuracy rose to 0.651 (a 0.219 increase) (Table S2). Some beginners reached accuracy levels comparable to experts, though variations in skill and expertise influenced individual improvement. Given that AC mechanisms assessment requires significant clinical experience, the model serves as a valuable educational tool, enabling beginners to efficiently learn from numerous UBM images and enhance their accuracy, thus benefiting ophthalmic training.

This study has some limitations. First, the model analyzed AC mechanisms in one single UBM image rather than comprehensively across one patient’s images, an area for future research. Second, while external testing showed promising results, the model was trained exclusively on images from one single UBM device, which may differ from devices in other hospitals. Expanding recognition to other UBM device brands could be achieved through transfer learning. Finally, due to the limited number of images, other AC mechanisms, such as plateau iris, were not identified. Although plateau iris is attributable to ALCB [24], modeled in this study, future work will involve collecting additional images to refine and enhance the model.

## 5. Conclusions

In conclusion, this study demonstrates ACM-Assessor’s effectiveness for accurately assessing AC mechanisms in UBM images. The system enables faster, more precise analyses and shows promise for use in primary and community hospitals with limited ophthalmic resources. Additionally, it holds potential as a training tool, helping beginners quickly enhance their UBM skills.

## Figures and Tables

**Table 1:** Comparison of the performance of classification models.

Classification	Algorithm	Accuracy (95% CI)	Sensitivity (95% CI)	Specificity (95% CI)	PPV (95% CI)	NPV (95% CI)	F1 Score	MCC	AUC
PB /Non-PB	RF	0.921 (0.901–0.937)	0.900 (0.863–0.928)	0.935 (0.910–0.954)	0.902 (0.866–0.930)	0.933 (0.909–0.952)	0.901	0.835	0.970
	GBDT	0.916 (0.895–0.932)	0.891 (0.854–0.920)	0.932 (0.906–0.951)	0.896 (0.859–0.925)	0.928 (0.903–0.948)	0.894	0.824	0.969
	XGB	0.916 (0.895–0.932)	0.889 (0.851–0.918)	0.933 (0.908–0.952)	0.899 (0.862–0.927)	0.927 (0.901–0.946)	0.894	0.824	0.970
	SVM	0.924 (0.905–0.940)	0.900 (0.863–0.928)	0.941 (0.917–0.958)	0.910 (0.874–0.936)	0.934 (0.909–0.952)	0.905	0.842	0.970
	LR	0.921 (0.901–0.937)	0.878 (0.839–0.909)	0.950 (0.927–0.966)	0.920 (0.886–0.946)	0.921 (0.895–0.941)	0.899	0.835	0.971
TPI /Non-TPI	RF	0.915 (0.890–0.934)	0.843 (0.792–0.883)	0.963 (0.938–0.978)	0.938 (0.897–0.963)	0.902 (0.868–0.927)	0.888	0.822	0.958
	GBDT	0.915 (0.890–0.934)	0.831 (0.780–0.873)	0.970 (0.947–0.984)	0.949 (0.910–0.972)	0.896 (0.863–0.922)	0.886	0.823	0.964
	XGB	0.922 (0.898–0.941)	0.854 (0.805–0.893)	0.968 (0.944–0.982)	0.946 (0.907–0.970)	0.908 (0.876–0.933)	0.898	0.838	0.970
	SVM	0.925 (0.902–0.943)	0.861 (0.813–0.899)	0.968 (0.944–0.982)	0.947 (0.908–0.970)	0.913 (0.881–0.937)	0.902	0.844	0.971
	LR	0.922 (0.898–0.941)	0.854 (0.805–0.893)	0.968 (0.944–0.982)	0.946 (0.907–0.970)	0.908 (0.876–0.933)	0.898	0.838	0.963
ALCB /Non-ALCB	RF	0.947 (0.924–0.964)	0.895 (0.837–0.935)	0.972 (0.948–0.986)	0.939 (0.888–0.969)	0.951 (0.923–0.970)	0.917	0.879	0.977
	GBDT	0.921 (0.894–0.942)	0.826 (0.759–0.878)	0.967 (0.941–0.982)	0.922 (0.965–0.957)	0.921 (0.888–0.945)	0.871	0.817	0.973
	XGB	0.938 (0.913–0.956)	0.884 (0.824–0.926)	0.964 (0.938–0.980)	0.921 (0.866–0.956)	0.946 (0.916–0.966)	0.902	0.857	0.972
	SVM	0.880 (0.849–0.906)	0.645 (0.568–0.716)	0.992 (0.974–0.998)	0.974 (0.919–0.993)	0.854 (0.816–0.886)	0.776	0.726	0.954
	LR	0.921 (0.894–0.942)	0.820 (0.752–0.873)	0.970 (0.945–0.984)	0.928 (0.871–0.962)	0.919 (0.885–0.943)	0.870	0.817	0.952

PB, pupillary block; TPI, thick peripheral iris; ALCB, anterior located ciliary body; RF, random forest; GBDT, gradient boosting decision tree; XGB, extreme gradient boosting; SVM, support vector machines; LR, logistic regression; CI, confidence interval; PPV, positive predictive value; NPV, negative predictive value; MCC, Matthews correlation coefficient.

**Table 2:** Performance of the model and ophthalmologists.

	Accuracy of Classification (95% CI)				Times (s)
	PB /Non-PB	TPI /Non-TPI	ALCB /Non-ALCB	The Integrated Assessment	
model	0.867 (0.831–0.897)	0.828 (0.789–0.862)	0.897 (0.863–0.923)	0.839 (0.800–0.872)	784.80
expert 1	0.812 (0.771–0.867)	0.888 (0.853–0.915)	0.867 (0.831–0.897)	0.729 (0.685–0.770)	2940.02
expert 2	0.837 (0.798–0.870)	0.755 (0.711–0.793)	0.862 (0.826–0.893)	0.798 (0.757–0.834)	3360.50
expert 3	0.929 (0.900–0.950)	0.853 (0.816–0.884)	0.908 (0.876–0.933)	0.849 (0.811–0.880)	2760.66
expert average	0.860 (0.823–0.891)	0.833 (0.794–0.866)	0.878 (0.843–0.907)	0.791 (0.750–0.828)	3020.39

PB, pupillary block; TPI, thick peripheral iris; ALCB, anterior located ciliary body; CI, confidence interval.

## Data Availability

Data are unavailable due to ethical restrictions.

## Supplementary Materials

The following supporting information can be downloaded at: https://www.mdpi.com/article/10.3390/bioengineering12040415/s1, Figure S1: The representative images of labeling PB and non-PB; Figure S2: The representative images of labeling TPI and non-TPI; Figure S3: The representative images of labeling ALCB and non-ALCB; Figure S4: The representative images of the integrated assessment; Table S1: The average performance changes in binary classification by the eight beginners before and after model assistance; Table S2: The changes in accuracy for integrated assessment by the beginners before and after model assistance; Table S3: Inter-expert agreement assessment during annotation.
