# Supplementary material for: ACM-Assessor: An Artificial Intelligence System for Assessing Angle Closure Mechanisms in Ultrasound Biomicroscopy
Source: Bioengineering (Basel). 2025 Apr 14;12(4):415. doi: 10.3390/bioengineering12040415 (PMC12025151; doi:10.3390/bioengineering12040415)
Supplement: Supplementary file 1 [file bioengineering-12-00415-s001.zip › bioengineering-3512741-supplementary.pdf]

Table S1. The average performance changes in binary classification by the eight beginners before and after model assistance.

	**Accuracy**		**Sensitivity**		**Specificity**		**PPV**		**NPV**	
	before	after	before	after	before	after	before	after	before	after
PB/Non-PB	0.706	0.817	0.663	0.763	0.727	0.844	0.543	0.734	0.815	0.873
TPI/Non-TPI	0.696	0.827	0.522	0.713	0.760	0.869	0.471	0.681	0.808	0.889
ALCB/Non-ALCB	0.704	0.814	0.669	0.692	0.745	0.912	0.648	0.873	0.748	0.786

PB, pupillary block; TPI, thick peripheral iris; ALCB, anterior located ciliary body; CI, confidence interval; PPV, positive predictive value; NPV, negative predictive value.

Table S2. The changes in accuracy for integrated assessment by the beginners before and after model assistance.

	Accuracy	
	before	after
beginner 1	0.264	0.665
beginner 2	0.489	0.624
beginner 3	0.445	0.647
beginner 4	0.750	0.821
beginner 5	0.337	0.415
beginner 6	0.420	0.617
beginner 7	0.369	0.748
beginner 8	0.381	0.667
average	0.432	0.651

Table S3. Inter-expert agreement assessment during annotation.

	agreement rate	kappa
PB/non-PB	0.928	0.855
TPI/non-TPI	0.902	0.802
ALCB/non-ALCB	0.917	0.809
The integrated assessment	0.862	0.812

PB, pupillary block; TPI, thick peripheral iris; ALCB, anterior located ciliary body.

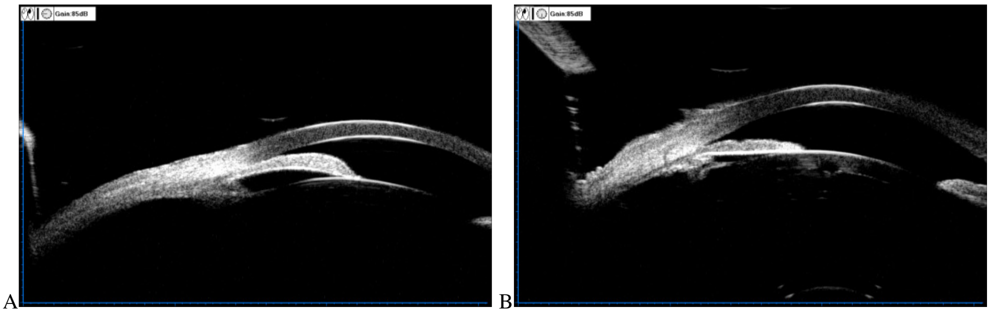

Figure S1. The representative images of labeling PB and non-PB.

According to their clinical experience and definition[7], [8], [18], [27] (convex forward iris profile, giving the typical bombe appearance, a very small zone of iris-lens contact in the center and shallow peripheral anterior chamber). PB, pupillary block.

(A)Representative images of PB

(B)Representative images of non-PB

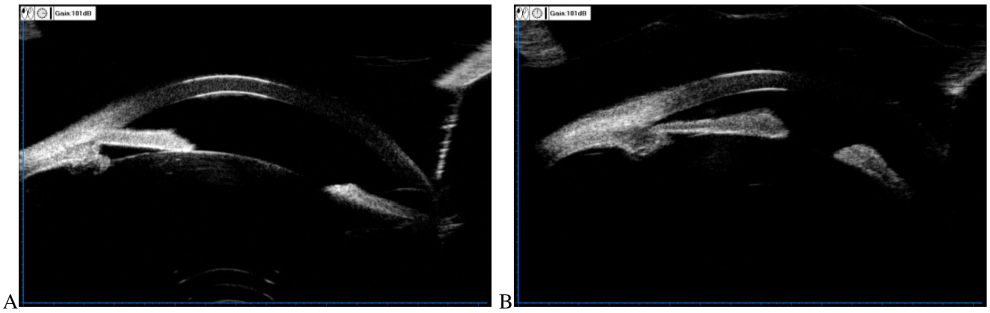

Figure S2. The representative images of labeling TPI and non-TPI.

According to their clinical experience and definition[7], [8], [18], [27] (thick peripheral iris occupying much of the angle, relatively deep central anterior chamber with shallow periphery). TPI, thick peripheral iris.

(A)Representative images of TPI

(B)Representative images of non-TPI

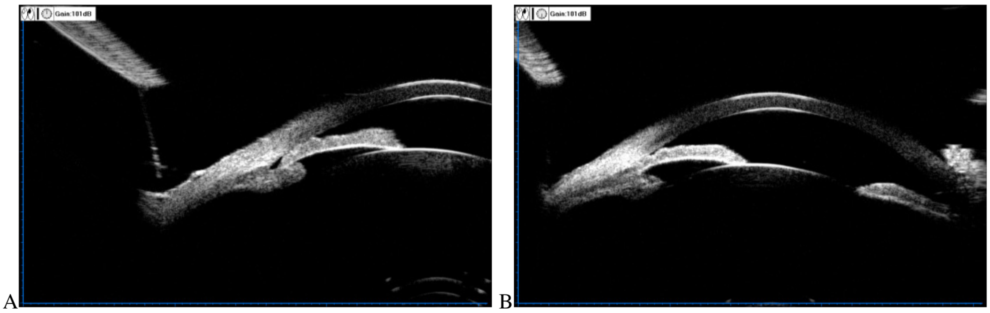

Figure S3. The representative images of labeling ALCB and Non-ALCB.

According to their clinical experience and definition[7], [8], [18], [27] (extensive contact between the ciliary body and the iris root, unobservable ciliary sulcus). ALCB, anterior located ciliary body.

(A)Representative images of ALCB

(B)Representative images of non-ALCB

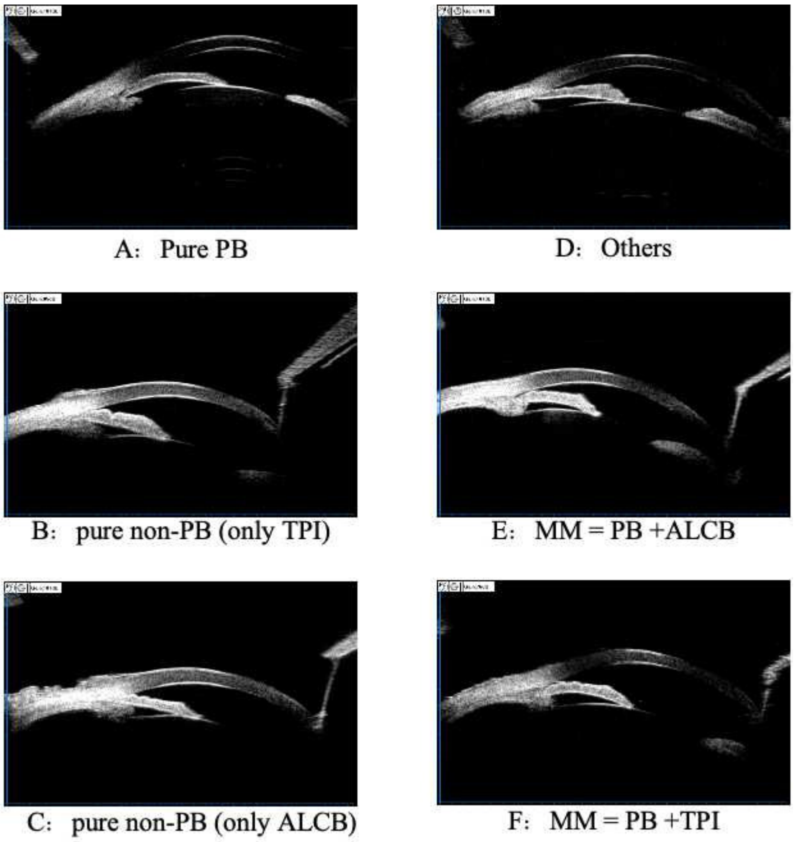

Figure S4. The representative images of the integrated assessment.

PB, pupillary block; TPI, thick peripheral iris; ALCB, anterior located ciliary body; MM, multiple mechanisms.
